# Numerical investigation of Al_2_O_3_/water nanofluid laminar convective heat transfer through triangular ducts

**DOI:** 10.1186/1556-276X-6-179

**Published:** 2011-02-28

**Authors:** Saeed Zeinali Heris, Seyyed Hossein Noie, Elham Talaii, Javad Sargolzaei

**Affiliations:** 1 Chemical Engineering Department, Faculty of Engineering, Ferdowsi University of Mashhad, Mashhad, Iran

## Abstract

In this article, laminar flow-forced convective heat transfer of Al_2_O_3_/water nanofluid in a triangular duct under constant wall temperature condition is investigated numerically. In this investigation, the effects of parameters, such as nanoparticles diameter, concentration, and Reynolds number on the enhancement of nanofluids heat transfer is studied. Besides, the comparison between nanofluid and pure fluid heat transfer is achieved in this article. Sometimes, because of pressure drop limitations, the need for non-circular ducts arises in many heat transfer applications. The low heat transfer rate of non-circular ducts is one the limitations of these systems, and utilization of nanofluid instead of pure fluid because of its potential to increase heat transfer of system can compensate this problem. In this article, for considering the presence of nanoparticl: es, the dispersion model is used. Numerical results represent an enhancement of heat transfer of fluid associated with changing to the suspension of nanometer-sized particles in the triangular duct. The results of the present model indicate that the nanofluid Nusselt number increases with increasing concentration of nanoparticles and decreasing diameter. Also, the enhancement of the fluid heat transfer becomes better at high Re in laminar flow with the addition of nanoparticles.

## Introduction

The increase of heat transfer coefficient is one of the most important technical aims for industry and researches. Also, the decrease in the pressure drop for systems that generate high fluid pressure drop is very noticeable. The aim, therefore, for achieving the optimization of heat exchangers must be always to increase the heat transfer, and simultaneously minimize the increase in the pressure drop [1]. Increased efforts are being directed to produce heat exchangers with higher efficiency to achieve savings of energy, material, and labor [2]. Improvements in heat transfer augmentation depend on performance and manufacturing cost [3]. Consequently, there is an increased need for utilization of a variety of duct geometries for heat transfer applications with forced convection and internal flow [2]. Because of the size and volume constraints in applications, such as aerospace, nuclear, biomedical engineering, and electronics, the utilization of non-circular flow passage geometries may be required, particularly, in respect of compact heat exchangers [2]. Consequently, duct with non-circular cross section (triangular) is used in this study because of its low pressure drop, but it causes decrement of heat transfer. Therefore, for compensating this decrement, nanofluid, instead of pure fluid, was used in this study because of the former's potential to increase the heat transfer of the system. Nanofluids are created by dispersing nanometer-sized particles (<100 nm) in a base fluid such as water, ethylene-glycol, or propylene-glycol [4].

Choi [5] was the first person to have created fluids containing suspension of nanometer-sized particles which are called the nanofluids and disclosed their significant thermal properties through the measurement of the convective heat transfer coefficient of those fluids. Various benefits of the application of nanofluids, such as improved heat transfer, size reduction of the heat transfer system, minimal clogging, microchannel cooling, and miniaturization of systems, were achieved in his study. Since then investigations have been continued at three phases as described below:

1. Conductive heat transfer was investigated in studies by many researchers, e.g., Lee et al. [6], which include measurement of conductive heat transfer coefficients of Al_2_O_3_/water, Al_2_O_3_/ethylene-glycol, CuO/water, and CuO/ethylene-glycol nanofluids.

2. Convective heat transfer was also studied in some published articles. For example, Pak and Cho [7] investigated convective heat transfer in the turbulent flow regime using Al_2_O_3_/water and TiO_2_/water nanofluids, and found that the Nusselt number of the nanofluids increased with increasing volume fraction of the suspended nanoparticles, and the increasing Reynolds number. Lee and Choi [8] studied convective heat transfer of laminar flows of an unspecified nanofluid in microchannels, and observed a reduction in thermal resistance by a factor of 2. Nanofluids were also observed to have the ability to dissipate a heat power three times more than pure water could do. Xuan and Li [9] measured convective heat transfer coefficient of Cu/water nanofluids, and found substantial heat transfer enhancement. For a given Reynolds number, heat transfer coefficient of nanofluids containing 1% volume Cu nanoparticles was shown to be approximately 12% higher than that of pure water.

Zienali Heris et al. [10-12] investigated the convective heat transfer of Al_2_O_3_/water and CuO/water nanofluids in circular tubes, and observed that the heat transfer coefficient was enhanced by increasing the concentration of nanoparticles in the nanofluids. However, the 20-nm Al_2_O_3 _nanoparticles showed an improved heat transfer performance compared with the 50-nm CuO nanoparticles, especially at high concentrations.

Maiga et al. [13] studied numerically the heat transfer enhancement in turbulent tube flow using Al_2_O_3 _nanoparticles suspension. Their results showed that the inclusion of nanoparticles into the base fluid produced an augmentation of the heat transfer coefficient which has been found to increase appreciably with an increase in the concentration of particles.

Akbari and Behzadmehr [14] investigated the developing laminar-mixed convection flow of a nanofluid consisting of Al_2_O_3_/water in a horizontal tube and hypothesized that the nanoparticles' concentration did not have any significant effect on the secondary flow pattern and the axial velocity.

Das and Ohal [15] studied numerically the behavior of nanofluids inside a partially heated and partially cooled square cavity to gain insight into heat transfer and flow processes induced by a nanofluid. Ben Mansour et al. [16] investigated the conjugate problem of developing laminar-mixed convection flow and heat transfer of Al_2_O_3_/water nanofluid inside an inclined tube subjected to a uniform wall heat flux. The Cu/water nanofluid-forced convective heat transfer performance in a circular tube was experimentally investigated by Zeinali Heris et al. [17]. Based on their experimental results, they observed that the heat transfer coefficient was influenced by Peclet number, as well as by Cu nanoparticles' volume concentrations. They also stated that there was an optimum concentration for Cu nanoparticles in water, in which improved enhancement for heat transfer can be found [17].

3. Boiling heat transfer: The boiling process of nanofluids was investigated experimentally by several researchers [18-21]. Das et al. [18] observed the nanofluids' boiling performance deterioration. Soltani et al. [19], through experimental measurements of boiling heat transfer characteristics of Al_2_O_3_/water and SnO_2_/water Newtonian nanofluids, showed that nanofluids possess noticeably higher boiling heat transfer coefficients than those of the base fluid.

Bang and Chang [21] studied boiling heat transfer characteristics of nanofluids with alumina nanoparticles suspended in water. They found that the addition of alumina nanoparticles caused a decrease of pool nucleate boiling heat transfer.

Noie et al. [22] investigated heat transfer enhancement using Al_2_O_3_/water nanofluids in a two-phase closed thermosyphon. Experimental results showed that for different input powers, the efficiency of the TPCT increases up to 14.7% when Al_2_O_3_/water nanofluid was used instead of pure water. The comparison between heat transfer enhancements using metallic and oxide nanoparticles was done by Hamed Mosavian et al. [23] whose results indicated the enhancement of heat transfer with increasing nanoparticles. Based on their experimental results, metallic nanoparticles showed better enhancement of heat transfer coefficient in comparison with oxide particles.

There are many passive cases regarding nanofluids that are still unrecognized. The most of the investigations were regarding heat transfer in circular ducts, and there is no report regarding ducts with a triangular cross section which causes lower pressure drop than the other forms of ducts. Different criteria for selecting and optimizing the heat exchanger passage geometries were outlined by Bergles [24]. Kays and London [25] showed that a compact heat exchanger, with a triangular cross-sectional internal flow passage, had a high ratio of heat transfer area to flow-passage volume. As far back as the late 1950s and early 1960s, Eckert et al. [26], Sparrow [27], Sparrow and Haji-Sheikh [28], and Schmidt and Newell [29] used approximate solution methods to study the pressure drop and convective heat transfer in fully developed laminar flow in ducts with cross sections having an equilateral or isosceles triangular section. Shah [30] and Shah and London [31] studied the heat transfer characteristics of laminar flow in a wide variety of channel shapes, including equilateral triangular, equilateral triangular with rounded corners, isosceles triangular, right triangular, and arbitrary triangular cross-sectional ducts, for an extensive range of thermal boundary conditions. Recently, Zhang [32] has reported Nusselt numbers for laminar hydrodynamically fully developed and thermally developing flow for a uniform wall temperature condition in isosceles triangular ducts with apex angles ranging from 30° to 120°. Gupta et al. [33] studied fully developed laminar flow and heat transfer in equilateral triangular cross-sectional ducts following serpentine and trapezoidal path. Kuznetsov et al. [34] studied the effects of thermal dispersion and turbulence in forced convection in a composite parallel-plate channel and stated: "Although the flow in the porous region remains laminar, thermal dispersion may have a dramatic impact on heat transfer in the channel."

The aim of this article is to study the laminar-forced convective flow heat transfer of nanofluid in a triangular duct with constant wall temperature using the dispersion model.

### Mathematical modeling

**Figure 1:**
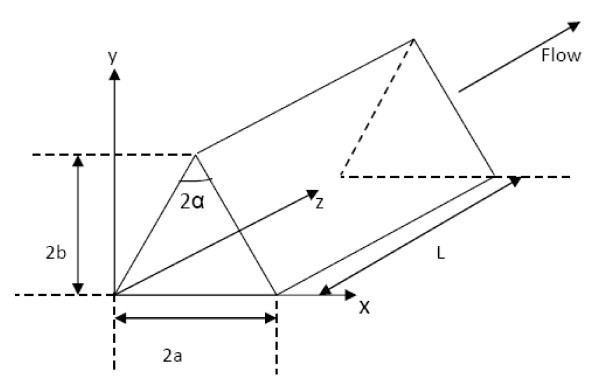
**Geometry of a triangular duct**.

Laminar flow-forced convection of Al_2_O_3_/water nanofluid in a triangular duct is studied numerically. The duct configurations and coordinate system are shown in Figure 1.

The assumptions of the model presented in this article can be summarized as follows:

1. Fully developed, steady-state, laminar flow.

2. Constant wall temperature.

3. Neglecting the axial diffusion terms in the equations of conservation of momentum and energy.

4. Neglecting *x*, *y *direction convective terms.

5. Equilateral triangular duct considered for investigation.

6. Neglecting viscous dissipations in Cartesian coordinates

For the hydrodynamically developed and thermally developing flow, there is only one nonzero component of velocity (*u*), and the constitutive equations of motion reduce to a single nonlinear partial differential equation of the form:

(1) $$\frac{\partial }{\partial x}\left(\mu \frac{\partial u}{\partial x}\right)+\frac{\partial }{\partial y}\left(\mu \frac{\partial u}{\partial y}\right)=\frac{\text{d}p}{\text{d}z}$$

The dimensionless parameters are defined as follows:

(2) $$Z=\frac{z}{2a}$$

(3) $$Y=\frac{y}{2b}$$

(4) $$X=\frac{x}{2a}$$

(5) $$\theta =\frac{T-T_{\text{w}}}{T_{\text{i}}-T_{\text{w}}}$$

(6) $$u^{*}=\frac{-\mu u}{\left(\frac{\text{d}p}{\text{d}Z}\right)D_{\text{h}}^{2}}$$

The dimensionless momentum equation can be written as [32]

(7) $$\frac{\partial^{2}u^{*}}{\partial X^{2}}+{\left(\frac{a}{b}\right)}^{2}\frac{\partial^{2}u^{*}}{\partial Y^{2}}+\frac{4a^{2}}{D_{\text{h}}^{2}}=0$$

Consequently, the dimensionless velocity profile for equilateral triangular duct defining $U=\frac{u}{u_{m}}$ is calculated as [31]

(8) $$U=\frac{15}{b^{2}}\left[-b^{2}Y^{3}+3a^{2}YX+\left(b^{2}Y^{2}+a^{2}X^{2}\right)-\left(\frac{4}{27}\right)b^{2}\right]$$

On the basis of the above assumptions, the energy equation for constant property flow is defined as

(9) $$\frac{k_{\text{eff}}}{\rho Cp}\left(\frac{\partial }{\partial x}\left(\frac{\partial T}{\partial x}\right)+\frac{\partial }{\partial y}\left(\frac{\partial T}{\partial y}\right)\right)=u\frac{\text{d}T}{\text{d}z}$$

where *k*_eff _is the effective thermal conductivity of the nanofluid. There are different approaches that are discussed in the literature for investigating the heat transfer of nanofluids. In the first approach (homogeneous model), the flow and energy equations of the base fluid are not affected by the presence of the nanoparticles. Under this assumption, both the fluid phase and solid nanoparticles flow at the same velocity and are in thermal equilibrium [35,36].

In the second approach, for the contribution of hydrodynamic dispersion and irregular movement of the nanoparticles, modified homogeneous model or dispersion model is adopted [35,36].

In this investigation, the dispersion model, in which the effect of random movement of nanoparticles inside the liquid is considered as excess terms in the heat transfer equation, is solved. The effective thermal conductivity of the nanofluid may take the following form [35]:

(10) $$k_{\text{eff}}=k_{\text{nf}}+k_{\text{d}}$$

where *k*_d _is the dispersion thermal conductivity. With respect to the similarity between diffusion in porous media and nanofluid flow, the following formula has been proposed to calculate *k*_d _[37-39]:

(11) $$k_{\text{d}}=c{\left(\rho Cp\right)}_{\text{nf}}u_{m}vd_{\text{p}}a$$

where (*c*) is an unknown constant, and should be determined by matching experimental data. It depends on the diameter of the nanoparticles and flowing surface geometry. The comparison of the measured values of the nanofluid thermal conductivity with the calculated values from the proposed models indicates that the thermal dispersion is the main mechanism for enhancing fluid thermal conductivity inside channels filled with nanofluids under convective conditions. In fact, Equation 11 is a first approximation considering the dispersive effects of nanoparticles on the thermal conductivity of the nanofluid flowing through channels. According to the study of Khaled and Vafai [40] in which heat transfer of nanofluid flow in a channel was investigated, the range of the value of c was chosen to be from 0 to 0.4. Comparing this study (triangular duct) with the channel flow in the Khaled and Vafai's investigation, the value of *c *= 0.3 is used in this study. In order to examine the exact value of constant (*c*), further experimental and numerical investigations are needed.

Finally, the energy equation for laminar flow in an equilateral triangular duct is

(12) $$2aU\frac{\partial \theta }{\partial Z}=\left[\frac{k_{\text{nf}}}{{\left(\rho Cp\right)}_{\text{nf}}u_{m}}+\frac{c{\left(\rho Cp\right)}_{\text{nf}}vd_{\text{p}}a}{{\left(\rho Cp\right)}_{\text{nf}}}\right]\frac{\partial^{2}\theta }{\partial X^{2}}+{\left(\frac{a}{b}\right)}^{2}\left[\frac{k_{\text{nf}}}{{\left(\rho Cp\right)}_{\text{nf}}u_{m}}+\frac{c{\left(\rho Cp\right)}_{\text{nf}}vd_{\text{p}}a}{{\left(\rho Cp\right)}_{\text{nf}}}\right]\frac{\partial^{2}\theta }{\partial Y^{2}}$$

where Peclet number can be used for simplifying the equation

(13) $$Pe_{\text{nf}}=\frac{2au_{m}{\left(\rho Cp\right)}_{\text{nf}}}{k_{\text{nf}}}$$

Consequently, the temperature distribution equation takes the form:

(14) $$2aU\frac{\partial \theta }{\partial Z}=\left[\frac{2a}{Pe_{\text{nf}}}+cvd_{\text{p}}a\right]\frac{\partial^{2}\theta }{\partial X^{2}}+{\left(\frac{a}{b}\right)}^{2}\left[\frac{2a}{Pe_{\text{nf}}}+cvd_{\text{p}}a\right]\frac{\partial^{2}\theta }{\partial Y^{2}}$$

Dimensionless bulk temperature is defined in the following form [40]:

(15) $$\theta_{\text{b}}=\frac{\iint u^{*}\theta \;\text{d}A}{\iint u^{*}\;\text{d}A}$$

An energy balance in a control volume in the duct will give the equation for the estimation of the local Nusselt number as follows:

(16) $$Nu=\frac{-\left(\frac{\partial \theta }{\partial X}\right){\;}_{X=1}}{\theta_{\text{b}}}$$

### Thermophysical properties of nanofluids

The thermophysical properties of nanofluid in the Equations 13-14 were calculated from nanoparticles and water properties using the following correlation at the mean bulk temperature [5,35,41]:

(17) $${\left(\rho Cp\right)}_{\text{nf}}=(1-\nu ){\left(\rho Cp\right)}_{\text{f}}+\nu {\left(\rho Cp\right)}_{\text{s}}$$

Thermal conductivity is the most important parameter indicating the enhancement potential of the nanofluids. Based on the studies carried out to evaluate the thermal conductivity of the nanofluids [5,6,42-45], the theoretical models cannot predict the thermal conductivity of nanofluids. In the absence of experimental data, Yu and Choi's correlation [44] used for the determination of the nanofluid's effective thermal conductivity is as follows:

(18) $$k_{\text{nf}}=\left[\frac{k_{\text{s}}+2k_{\text{w}}+2(k_{s}-k_{\text{w}}){\left(1+\beta \right)}^{3}\nu }{k_{\text{s}}+2k_{\text{w}}-(k_{\text{s}}-k_{\text{w}}){\left(1+\beta \right)}^{3}\nu }\right]k_{\text{w}}$$

where *β *is the ratio of the nanolayer thickness to the original particle radius, and *β *= 0.1 was used to calculate the nanofluid's effective thermal conductivity.

### Validation of the simulation

In this article, the finite difference method is used for numerical solution. The discretization in the physical space (*x*, *y*) is performed by dividing the flow domain in equal triangular elements. The grid is constructed by drawing inside the triangular cross section three groups of parallel lines. The lines of each group are equally distanced and parallel to one of the three sides of the triangle.

**Figure 2:**
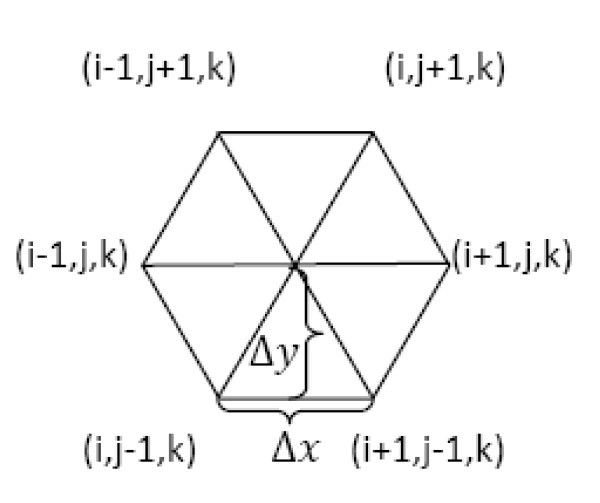
**Hexagonal computational cell and finite differencing along a characteristic**.

The benefit of such a discretization is obvious. Since the boundaries of the computational domain are identical to the boundaries of the triangular cross sections of the channel, this method provides good accuracy in the numerical solution [45,46]. Hexagonal computational cell and finite differencing along a characteristic is shown in Figure 2.

Equation 14 was discretized using central differencing for $\left(\frac{\partial^{2}\theta }{\partial Y^{2}}\right)$ and $\left(\frac{\partial^{2}\theta }{\partial X^{2}}\right)$ and backward differencing for $\left(\frac{\partial \theta }{\partial Z}\right)$

Boundary conditions in dimensionless form read as follows:

(19) θ(i,j,1)=1

(20) θ(1,j,k)=0

(21) θ(i,1,k)=0

(22) θ(i,m,k)=0

(23) $$\frac{\partial \theta }{\partial x}\left(\frac{m+1}{2},j,k\right)=0$$

where *m *is gride number in *x *and *y *directions. The grid used in the present analysis is 560 × 560 × 100 nonuniform one with highly packed grid points in the vicinity of the tube wall and especially in the entrance region (560 in *x*, *y *direction; 100 in *z *direction). In order to ensure grid independence, the solution is tested for the 800 × 800 × 180 and the 500 × 500 × 90 configurations, with all of the latter giving similar values. Therefore, 560 × 560 × 100 configuration was accepted as the optimal grid size.

**Figure 3:**
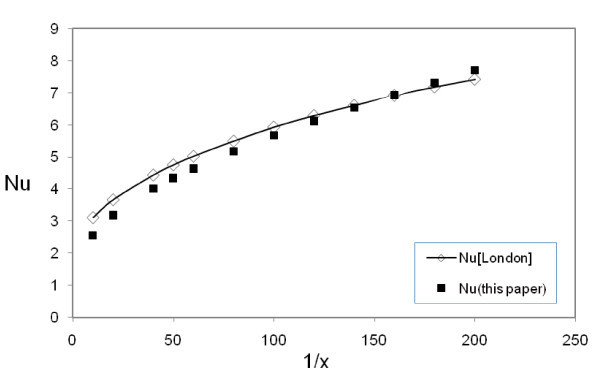
**Comparison between model predictions and results defined by Shah and London **[31].

The duct is an equilateral triangle with a length of each side being 100 cm. The flow is laminar with the Reynolds number ranging from 100 to 2100. Because of the absence of experimental data for nanofluid in triangular ducts, in order to validate the computational model, the numerical results were compared with the theoretical data available for the conventional fluids in triangular duct as proposed by Shah and London [31]. Figure 3 displays the comparison of Nusselt number values computed by Shah and London [31] with the computed values from the present simulations.

## Results and discussion

In this section, the effect of nanoparticle's diameter, nanoparticle's concentration, and Reynolds number on heat transfer performance of Al_2_O_3_/water nanofluid is investigated.

**Figure 4:**
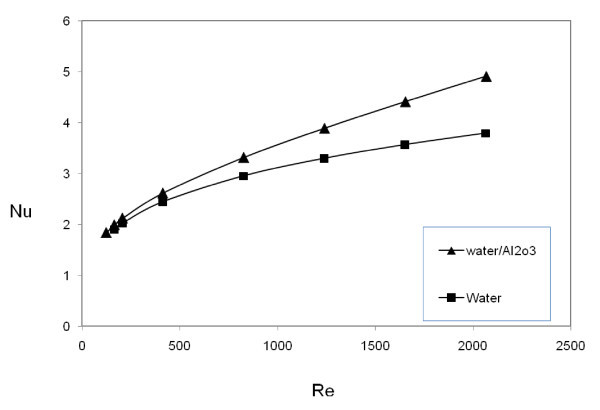
**Comparison between nanofluid and pure fluid heat transfer**.

Figure 4 shows the average Nusselt numbers versus Re for Al_2_O_3_/water nanofluid (with 1.0% volume concentration of 10-nm Al_2_O_3 _nanoparticles) and pure water. As shown in Figure 4, the slope of *Nu *versus *Re *is greater for Al_2_O_3_/water compared with pure water, which means a considerable enhancement of heat transfer due the addition of nanoparticles to the base fluid. For example, at *Re *= 1500, Nusselt number of water is increased from 3.47 to 4.22 with the addition of Al_2_O_3 _nanoparticles. It is known that the addition of Al_2_O_3 _nanoparticles will increase the thermal conductivity of the working fluid and hence the heat transfer capability [5-12]. Besides, the nanoparticles with dispersion effect and Brownian motion hit the tube wall and absorb heat, and then mix back with the bulk of the fluid to cause a better heat transfer. The presence of nanoparticles inside the fluid causes the collision between the heating surface and the particles, thereby producing higher heat transfer coefficients. This means that the addition of nanoparticles to fluid changes the flow structure so that besides the increase in thermal conductivity, dispersion and fluctuation of nanoparticles, especially near the tube wall, lead to the increase in the energy exchange rates and augment the heat transfer rate between the fluid and the tube wall [22,23]. Moreover, the local Nu in fluid flow inside channel is related to the thickness of the thermal boundary layer, and a decrease in thermal boundary-layer thickness increases local Nu. One of the possible mechanisms responsible for the exhibition of the thermal boundary-layer thickness decrement by nanofluid is the migration of the nanoparticles due to shear action, Brownian motion, and the viscosity gradient in the cross section of the channel [47].

**Figure 5:**
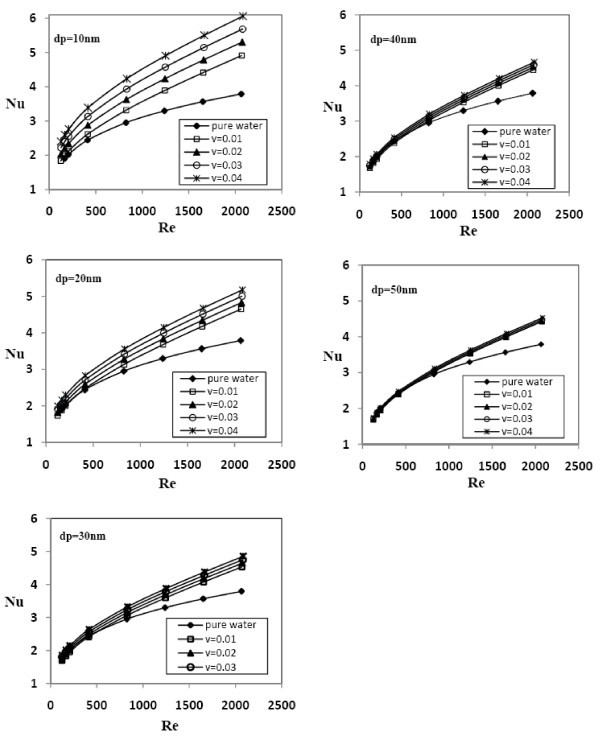
**The influence of Al_2_O_3 _nanoparticles' volume concentration on the Nusselt number over a range of Reynolds numbers with diameter of nanofluids in the range of 10-50 nm**.

Figure 5 shows the plots of the average Nusselt number versus *Re *at various concentrations of Al_2_O_3 _for 10-50 nm nanoparticles. This figure indicates that the average Nusselt number increases with the concentration of the nanoparticles, and better enhancement is seen at higher Reynolds numbers. For example, at d*p *= 10 and *Re *= 400, by increasing nanoparticle's concentration from 0.01 to 0.04, the average Nusselt number increases from 2.588 to 3.345, or at higher Reynolds number (*Re *= 2050), the Nusselt number changes from 4.89 to 6.02. The average Nusselt number at the same diameter increases according to Reynolds number. The results illustrate that by increasing Reynolds number from 500 to 2070 at d*p *= 30 nm, for 0.01 volume concentration of Al_2_O_3_/water nanofluid, the average Nusselt number increases from 2.57 to 4.53.

During the nanofluid flow through the channel, migration of the nanoparticles and clustering due to non-uniform shear rate across the channel's cross section affect the heat transfer performance. Taking into account the increase in thermal conductivity of the nanofluid, other factors such as chaotic movement of nanoparticles, Brownian motion, and particles' migration must also be considered in the interpretation of heat transfer performance of nanofluids [35]. An increase in the volume fraction of the nanoparticles intensifies the interaction and collision of the nanoparticles. Also, diffusion and relative movement of these particles near the channel walls lead to the rapid heat transfer from the walls to the nanofluid. In other words, increasing the concentration of the nanoparticles intensifies the mechanisms responsible for the enhanced heat transfer. Moreover, at high flow rates, the dispersion effects and chaotic movement of the nanoparticles intensify the mixing fluctuations and change the temperature profile to a flatter profile similar to turbulent flow and cause an increase in the heat-transfer coefficient. At low flow rates, clustering and agglomeration of nanoparticles may exist in the nanofluid flow, and therefore, at a low *Re*, a lower heat transfer enhancement can be observed.

**Figure 6:**
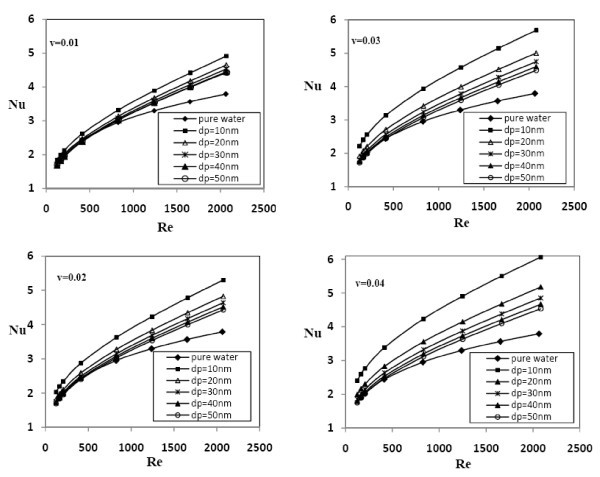
**Effect of nanoparticle's diameter on the Nusselt number for**. **(a) **1% volume concentration, **(b) **2% volume concentration, **(c) **3% volume concentration, and **(d) **4% volume concentration of Al_2_O_3 _nanofluids.

Figure 6 displays the effect of nanoparticle's diameter on the Nusselt number for Al_2_O_3 _nanofluids of constant volume concentrations. It can be seen that the average Nusselt number increases with the decreasing size of nanoparticles at the same concentration, particularly at high concentrations. For example, by increasing the size of the nanoparticles from 10 to 50 nm in 0.02 concentration at *Re *= 400, the average Nusselt numbers decrease from 2.845 to 2.376. Also, at Reynolds number 2050 in 0.02 volume concentration, increasing the nanoparticle's size from 10 to 50 nm leads to a decrease in *Nu *from 5.273 to 4.406. Similar kind of enhancement in Nusselt number with smaller particle size was observed from the experiments conducted by Zeinali Heris et al. [10].

Since heat transfer between the nanoparticles and the fluid takes place at the particle-fluid interface, the ratio of the surface area of nanoparticles to their volume is the most important factor for heat transfer enhancement by nanofluids. On decreasing the size of nanoparticles, the ratio of surface area-to-volume of nanoparticles increases, allowing them to absorb and transfer heat more efficiently. Moreover, for the particles with very small diameter, the particles' distribution is fairly uniform; on the other hand, by increasing the nanoparticles' mean diameter, non-uniformity on the particles' distribution becomes more important, and the nanoparticles' concentration becomes higher at the vicinity of wall for which the viscous forces are important; this causes a decrement in the heat transfer enhancement while using the nanofluid with large particle's size.

For laminar flow, the heat transfer coefficient is mainly proportional to the fluid's thermal conductivity. Also, as already mentioned, the main effects of nanoparticles inside the fluid considering the Brownian motion and fluctuation are the change in the flow structure of fluid to semi-turbulence regime, and flattening of the transverse temperature gradient in the bulk of the fluid [9], and hence enhancing convective heat transfer of the nanofluid. However, in the turbulent regime, this mechanism is not dominant, and the thermal conductivity increment is the only factor for heat transfer enhancement in turbulent flow. For the turbulent flow, as the heat transfer coefficient depends to a smaller degree on the thermal conductivity of the fluid, the effect of thermal conductivity becomes less pronounced, since, in the presence of the nanoparticles, the aforementioned heat transfer enhancement may be decreased for turbulent flow.

A detailed review of the literature revealed that it might be beneficial to avail the advantage (i.e., lower pressure drop) of using triangular cross-sectional ducts in thermal engineering systems. However, heating exchange rates will decrease through such conduits. On the contrary, the results of this preliminary numerical study revealed that this could be compensated by using nanofluids in these systems, and so this will enhance the heat transfer rates. The use of nano-sized solid particles additives suspended into the base fluid (nanofluids) is a technique recommended for the enhancement of heat transfer in the triangular ducts. There are very few correlations available to exactly predict the heat transfer performance of nanofluids, as well as correlations which include the effects of solid particles' concentration, shape, size, dispersion, and nanoparticles' random movement are not suffice. Therefore, further research on convective heat transfer of nanofluids, and more theoretical and experimental research studies are needed to clearly understand and accurately predict their hydrodynamic and thermal characteristics especially in triangular ducts.

## Conclusions

In this article, the laminar flow-forced convection of Al_2_O_3_/water nanofluid in a triangular duct is studied numerically. The results indicate that the addition of nanoparticles to base fluid, besides the thermal conductivity increment, affects the structure of the flow field and leads to heat transfer enhancement, because of the dispersion and random movement of nanoparticles inside the fluid. The results obtained by the numerical solutions show that decreasing the nanoparticle's size increases Nusselt number at a specific concentration, and increasing the nanoparticles' concentration increases Nusselt number at constant particle size. The results obtained in this preliminary study indicate that, in the case of using triangular cross-sectional ducts in thermal engineering systems, because of their low pressure drop, the decrement of heating exchange rate could be compensated by the use of nanofluids in these systems. Consequently, the flow of the nanofluids through triangular conduits has both the benefits of low pressure drop and high heat transfer rate.

### List of symbols

*d*_p_: Nanoparticles diameter (nm)

*k_d: _*Dispersion Thermal conductivity

*k*_w_: Thermal conductivity of water (W/mK)

*k*_s_: Thermal conductivity of solid nanoparticles (W/mK)

*k*_nf_: Thermal conductivity of nanofluid (W/mK)

*k*_eff_: Effective thermal conductivity (W/mK)

*Cp*_f_: Specific heat of fluid (kJ/kg K)

*Cp*_nf_: Specific heat of nanofluid (kJ/kg K)

*Cp*_s_: Specific heat of nanoparticles (kJ/kg K)

*Pe*_nf_: Peclet number of nanofluid

*T*: Nanofluid local temperature (K)

*T*_i_: Inlet temperature of nanofluid (K)

*T*_w_: Triangle wall temperature (K)

*u*: Local axial velocity(m/s)

$U=\frac{u}{u_{m}}$: dimensionless velocity profile*u_m_*: Average axial velocity (m/s)

u*: Dimensionless velocity

#### Greek letters

*θ*: Dimensionless temperature

*θ*_b_: Dimensionless bulk temperature

*ρ*_s_: Nanoparticles density (kg/m^3^)

*ρ*_f_: Fluid density (kg/m^3^)

*ρ*_nf_: Nanofluid density (kg/m^3^)

*μ*: Dynamic viscosity (kg/ms)

*v*: Volume fraction

## Competing interests

The authors declare that they have no competing interests.

## Authors' contributions

SZH planned the numerical investigation, took major part in the interpretation of results and participated in the manuscript preparation, SHN participated in the design of the study, ET drafted the manuscript, took part in the interpretation of results and participated in the manuscript preparation, and JS participated in the sequence alignment. All authors have read and approved the final manuscript.

## References

[B1] Tauscher R Mayinger F Heat transfer enhancement in a plate heat exchanger with rib-roughened surfaces 1998 Lehrstuhl afur Thermodynamik Technische Universitat Muchen, 85747 Garching, Germany

[B2] Sahin AZ Irreversibility's in various duct geometries with constant wall heat flux and laminar flow Energy 1998 23 6 465 473 10.1016/S0360-5442(98)00010-3

[B3] Kakac S Bergles AE Mayinger F Heat Exchangers. Thermal-Hydraulic Fundamentals and Design 1981 New York: McGraw-Hill

[B4] Namburu PK Das DK Tanguturi KM Vajjha RS Numerical study of turbulent flow and heat transfer characteristics of nanofluids considering variable properties Int J Thermal Sci 2009 48 290 302 10.1016/j.ijthermalsci.2008.01.001

[B5] Choi SUS Siginer DA Wang HP Enhancing thermal conductivity of fluid with nanoparticles Developments and Application of Non-Newtonian Flows 1995 66 New York: ASME 99 105

[B6] Lee S Choi SUS Li S Eastman JA Measuring thermal conductivity of fluids containing oxide nanoparticles J Heat Transf 1999 121 280 289 10.1115/1.2825978

[B7] Pak BC Cho YI Hydrodynamic and heat transfer study of dispersed fluids with submicron metallic oxide particles Exp Heat Transf 1999 11 151 170 10.1080/08916159808946559

[B8] Lee S Choi SUS Application of metallic nanoparticle suspensions in advanced cooling systems Proceeding of International Mechanical Engineering Congress and Exposition 1996 Atlanta, USA

[B9] Xuan Y Li Q Investigation on convective heat transfer and flow features of nanofluids J Heat Transf 2003 125 151 155 10.1115/1.1532008

[B10] Zeinali Heris S Etemad SGh Nasr Esfahany M Experimental investigation of oxide nanofluids laminar flow convective heat transfer Int Commun Heat Mass Transf 2006 33 529 533 10.1016/j.icheatmasstransfer.2006.01.005

[B11] Zeinali Heris S Nasr Esfahany M Etemad SGh Investigation of CuO/water nanofluid laminar convective heat transfer through a circular tube J Enhanc Heat Transf 2006 13 4 1 11

[B12] Zeinali Heris S Nasr Esfahany M Etemad SGh Experimental investigation of convective heat transfer of Al_2_O_3_/water nanofluid in circular tube Int J Heat Fluid Flow 2007 28 203 210 10.1016/j.ijheatfluidflow.2006.05.001

[B13] Maiga SB Nguyen CT Galanis N Roy G Mare T Coqueux M Heat transfer enhancement in turbulent tube flow using Al_2_O_3 _nanoparticle suspension Int J Numer Methods Heat Fluid Flow 2006 16 275 292 10.1108/09615530610649717

[B14] Akbari M Behzadmehr A Developing mixed convection of a nanofluid in a horizontal tube with uniform heat flux Int J Numer Methods Heat Fluid Flow 2007 17 566 586 10.1108/09615530710761216

[B15] Das MK Ohal PS Natural convection heat transfer augmentation in a partially heated and partially cooled square cavity utilizing nanofluids Int J Numer Methods Heat Fluid Flow 2009 19 411 431 10.1108/09615530910938353

[B16] Ben Mansour R Galanis N Nguyen CT Developing laminar mixed convection of nanofluids in an inclined tube with uniform wall heat flux Int J Numer Methods Heat Fluid Flow 2009 19 146 164 10.1108/09615530910930946

[B17] Zeinali Heris S Etemad SGh Nasr Esfahany M Convective heat transfer of a Cu/water nanofluid flowing through a circular tube Exp Heat Transf 2009 22 217 227 10.1080/08916150902950145

[B18] Das SK Putra K Roetzel W Pool boiling characteristics of nano-fluids Int J Heat Mass Transf 2003 46 5 851 862 10.1016/S0017-9310(02)00348-4

[B19] Soltani S Etemad SGh Thibault J Pool boiling heat transfer performance of Newtonian nanofluids Heat Mass Transf 2009 45 12 1555 1560 10.1007/s00231-009-0530-9

[B20] Vassallo P Kumar R Amico SD Pool boiling heat transfer experiments in silica-water nanofluids Int J Heat Mass Transf 2004 47 407 411 10.1016/S0017-9310(03)00361-2

[B21] Bang IC Chang SH Boiling heat transfer performance and phenomena of Al_2_O_3_/water nanofluids from a plain surface in a pool Int J Heat Mass Transf 2005 48 2407 2419 10.1016/j.ijheatmasstransfer.2004.12.047

[B22] Noie SH Zeinali Heris S Kahani M Nowee SM Heat transfer enhancement using Al_2_O_3_/water nanofluid in a two-phase closed thermosyphon Int J Heat Fluid Flow 2009 30 700 705 10.1016/j.ijheatfluidflow.2009.03.001

[B23] Hamed Mosavian MT Zeinali Heris S Etemad SGh Nasr Esfahany M Heat transfer enhancement by application of nano-powder J Nanoparticle Res 2010 12 2611 2619 10.1007/s11051-009-9840-6

[B24] Bergles AE Heat transfer enhancement-the encouragement and accommodation of high heat fluxes J Heat Transf 1997 119 8 19 10.1115/1.2824105

[B25] Kays WM London AL Compact Heat Exchangers 1984 New York: McGraw-Hill

[B26] Eckert ERG Irvine TF Yen JT Laminar heat transfer in wedge-shaped passage Trans ASME 1958 80 1433 1438

[B27] Sparrow EM Laminar flow in isosceles triangular ducts AICHE J 1962 5 599 604 10.1002/aic.690080507

[B28] Sparrow EM Haji-sheikh A Laminar heat transfer and pressure drop in isosceles triangular, right triangular and circular sector ducts ASME J Heat Transf 1964 87 426 427

[B29] Schmidt FW Newell ME Heat transfer in fully developed flow through rectangular and isosceles triangular ducts Int J Heat Mass Transf 1967 10 1121 1123 10.1016/0017-9310(67)90127-5

[B30] Shah RK Laminar flow friction & forced convection heat transfer in ducts of arbitrary geometry Int J Heat Mass Transf 1975 18 849 862 10.1016/0017-9310(75)90176-3

[B31] Shah RK London AL Laminar Flow Forced Convection in Ducts 1978 New York: Academic Press Inc

[B32] Zhang LZ Laminar flow and heat transfer in plate-fin triangular ducts in thermally developing entry region Int J Heat Mass Transf 2007 50 1637 1640 10.1016/j.ijheatmasstransfer.2006.09.013

[B33] Gupta RV Geyer PE Fletcher DF Haynes BS Thermohydraulic performance of a periodic trapezoidal channel with a triangular cross-section Int J Heat Mass Transf 2008 51 2925 2929 10.1016/j.ijheatmasstransfer.2007.09.017

[B34] Kuznetsov AV Cheng L Xiong M Effects of thermal dispersion and turbulence in forced convection in a composite parallel-plate channel: investigation of constant wall heat flux and constant wall temperature cases Numer Heat Transf A 2002 42 365 383 10.1080/10407780290059602

[B35] Zeinali Heris S Nasr Esfahany M Etemad SGh Numerical investigation of nanofluid laminar convective heat transfer through circular tube Numer Heat Transf A 2007 52 1043 1058 10.1080/10407780701364411

[B36] Xuan Y Rotzel W Conception for heat transfer correlation of nanofluid Int J Heat Mass Transf 2000 43 3701 3708 10.1016/S0017-9310(99)00369-5

[B37] Drew DA Passman SL Theory of Multi Component Fluids 1999 Berlin: Springer

[B38] Kaviany M Principles of Heat Transfer in Porous Media 1995 Berlin: Springer

[B39] Taylor GI Dispersion of soluble matter in solvent flowing through a tube Proc R Soc Lond 1954 A21 186

[B40] Khaled A-RA Vafai K Heat transfer enhancement through control of thermal dispersion effects Int J Heat Mass Transf 2005 48 2172 2185 10.1016/j.ijheatmasstransfer.2004.12.035

[B41] Akbarinia A Behzadmehr A Numerical study of laminar mixed convection of a nanofluid in horizontal curved tubes Appl Therm Eng 2007 27 1327 1337 10.1016/j.applthermaleng.2006.10.034

[B42] Eastman JA Choi SUS Li S Yu W Thomson LJ Anomalously increased effective thermal conductivities of ethylene glycol-based nanofluids containing copper nanoparticles Appl Phys Lett 2001 78 718 720 10.1063/1.1341218

[B43] Wang XQ Mujumdar AS A Review on nanofluids-part II: experiments and applications Brazilian J Chem Eng 2008 25 4 631 648

[B44] Yu W Choi SUS The role of interfacial layers in the enhanced thermal conductivity of nanofluids: a renovated Maxwell model J Nanoparticle Res 2003 5 167 171 10.1023/A:1024438603801

[B45] Jang SP Choi SUS Role of Brownian motion in the enhanced thermal conductivity of nanofluids Appl Phys Lett 2004 84 4316 4318 10.1063/1.1756684

[B46] Naris S Valougeorgis D Rarefied gas flow in a triangular duct based on a boundary fitted lattice Eur J Mech B 2008 27 810 822 10.1016/j.euromechflu.2008.01.002

[B47] Wen D Ding Y Experimental investigation into convective heat transfer of nanofluid at the entrance rejoin under laminar flow conditions Int J Heat Transf 2004 47 5181 5188 10.1016/j.ijheatmasstransfer.2004.07.012

